# Evaluation of Hypoglycemia and Associated Factors among Patients with Type 1 Diabetes on Follow-Up Care at St. Paul's Hospital Millennium Medical College, Addis Ababa, Ethiopia

**DOI:** 10.1155/2019/9037374

**Published:** 2019-04-10

**Authors:** Halefom Kahsay, Bereket Fantahun, Teshome Nedi, Gebre Teklemariam Demoz

**Affiliations:** ^1^Department of Pharmacy, College of Medicine and Health Sciences, Adigrat University, Adigrat, Ethiopia; ^2^Department of Pediatrics, St. Paul's Hospital Millennium Medical College, Addis Ababa, Ethiopia; ^3^School of Pharmacy, College of Health Sciences, Addis Ababa University, Addis Ababa, Ethiopia; ^4^School of Pharmacy, College of Health Sciences, Aksum University, P.O. Box: 298, Aksum, Ethiopia

## Abstract

**Background:**

Hypoglycemia is one of the most common acute complications of type 1 diabetes mellitus (T1DM). The knowledge of the factors associated with hypoglycemia will help in the prevention and management of the problem. Therefore, this study was conducted to assess hypoglycemia and its associated factors among T1DM patients who attended the diabetes outpatient clinic of St. Paul's Hospital Millennium Medical College (SPHMMC).

**Methods:**

A cross-sectional study was conducted at the diabetes clinic of SPHMMC. Data on sociodemographic and clinical characteristics including duration of diabetes, type of insulin they have been taking, the factors associated with hypoglycemia, and the severity stage of hypoglycemia was obtained. Data was collected using a structured questionnaire and chart review. Multivariate logistic regression model was used to identify factors associated with hypoglycemia.

**Result:**

Out of the 247 participants who were recruited into the study, 233 (94.3%) of them experienced hypoglycemia. A total of 6.9 events of hypoglycemia per patient per year happened. Particularly, the events were categorized as 3.1 mild events, 2.3 moderate events, and 0.93 severe events of hypoglycemia. Shorter duration of diabetes history (<1 year) was significantly associated with less experience of hypoglycemia (AOR = 0.09, 95% CI: 0.01-0.90). However, blood glucose monitoring at home was found to be significantly associated with more report of hypoglycemia (AOR = 5.77, 95% CI: 1.16-28.66).

**Conclusion:**

The prevalence of hypoglycemia among T1DM patients was found as substantially high. Self/family blood glucose monitoring at home could not guarantee to minimize the occurrence of hypoglycemia events. Finger stick home blood glucose monitoring should be given a special attention. Therefore, the involvement of health care providers in diabetes care should be encouraged to address the occurrence of hypoglycemia in T1DM patients.

## 1. Background

Hypoglycemia is the development of autonomic symptoms (trembling, palpitations, sweating, anxiety, hunger, and nausea) and tingling or neuroglycogenic symptoms [1–3]. Hypoglycemic episodes could also be categorized based on severity as mild, moderate, and severe [4, 5]. Hypoglycemia is a common problem in children with type 1 diabetes mellitus (T1DM) because of the challenges presented by insulin dosing, variable eating patterns, erratic activity, and the limited ability of small children to detect early signs of hypoglycemia [6–9]. The estimates of children and adolescents below age 19 with T1DM have risen to over a million [10]. Individuals who take insulin, which includes all people with T1DM and some people with type 2 diabetes, are prone to hypoglycemia [11].

Hypoglycemia is one of the most feared complications of diabetes treatment [12]. It commonly occurs in clinical practice as approximately 90% of all patients who receive insulin have experienced hypoglycemic episodes [13]. Furthermore, surveys investigating the prevalence of hypoglycemia have provided some alarming results. The Diabetes Control and Complication Trial (DCCT) reported a threefold increase in severe hypoglycemia and coma in intensively treated T1DM patients versus conventionally treated patients [14]. A population-based study conducted in the United Kingdom that determined the frequency and predictors of hypoglycemia in type one diabetic patients found that an intensively treated individual with T1DM can experience up to 10 episodes of symptomatic hypoglycemia per week and severe temporarily disabling hypoglycemia at least once a year [15]. A variety of figures were reported for the prevalence of hypoglycemia with similar studies in Taiwan (36.5%), Scotland (98%), South Africa (56%), and Nigeria (65%) [16–19].

Knowledge regarding the different factors that may influence hypoglycemia in T1DM is expanding. Many factors, including sociodemographic and clinical characteristics and treatment-related factors, have been reported in different literatures. For example, a population-based prospective cohort study done in the United States of America to determine risk factors for frequent and severe hypoglycemia in T1DM patients found in their multivariable analysis that lower HbA1c, intensive insulin therapy, and more frequent (3-4 times per day) SMBG were associated with increased risk of frequent (mild and moderate) hypoglycemic reactions whereas only lower HbA1C and older age were associated with severe hypoglycemic reactions [7]. On the other hand, a case control study in the Republic of Korea revealed that independent risk factors of severe hypoglycemia were associated with a lack of SMBG and previous episodes of severe hypoglycemia in addition to the positive association report with patients of older age and had history of prolonged duration of hypoglycemia [20].

Generally, this study has obtained the magnitude of hypoglycemia with its possible contributing factors. Besides, the severity of hypoglycemia has been investigated and measurements taken as immediate management during the occurrence of hypoglycemia have been identified. Thus, it could serve as an input for further studies on the prevalence of hypoglycemia and possible associated factors. Additionally, it encourages advanced home care self-management of hypoglycemia events and educational programs for health care providers.

## 2. Methods

This cross-sectional study was conducted to assess the prevalence and associated factors for hypoglycemia among T1DM patients attending regular follow-up at the diabetes clinics of SPHMMC. A total of 45 T1DM patients were seen weekly. Patient interview and chart review for the same patients were conducted from the 1st of June to the 1st of August 2018. Study population was selected among all T1DM patients on follow-up at the study area during the study period who fulfilled the inclusion criteria. Patients who were 5 years old and above and who were diagnosed with T1DM were eligible for inclusion while T1DM patients who were pregnant, with other chronic comorbidities (cirrhosis and heart and kidney failure infectious diseases), and with incomplete/inaccessible medical chart were excluded.

The sample size (*n*) was calculated assuming a 50% proportion (*p*) of hypoglycemia prevalence, a 5% marginal error (*d*), and a confidence interval (CI) of 95%. Based on this assumption, the sample size was calculated by a single population proportion formula (*n* = *Z*(*α*/2)^2^(*p*(1 − *p*)/*d*^2^)). This yields a sample size of 384. The expected number of patients in the study period was the source population (*N*). The number of patients with TDM who expected to visit the diabetes clinic during the diabetic days of the study period were 540. It was calculated by the total sum of 12∗10, 12∗10, and 12∗5 for those who were averagely attending every Wednesday and Thursday working hours and Friday morning, respectively. Additionally, it was also calculated as 12∗20 for those who attend every Monday afternoon. The sample size was thus adjusted and calculated using the following correction formula: Corrected sample size = (*n* × *N*/*n* + *N*)~225.

Due to the fact that there is a nonresponse (with an acceptable level of 10%), 10% of the calculated sample size was added to the estimated sample size making the final sample size of 248. A systematic random sampling technique was used to recruit patients for the study in each day of the data collection process. The actual sampling fraction (*k*^th^) was calculated by dividing the total number of source population attending during the study period (540) to the corrected sample size (248). Thus, every other patient was interviewed after physician visit and his/her medical record was reviewed in the same day after the interview until the total sample size was reached. Six nurses who had taken a one-day training regarding the objective, relevance, confidentiality, respondent's right, informed consent, and techniques of interview for the study using the structured questionnaire prior to data collection were recruited for data collection. Data was collected using interview supported by structured questionnaire which was originally prepared in English then translated to the local language, Amharic, which had three components.

The data obtained from pediatric T1DM patients who were eligible (5-18 years old) was acquired by interview with the respective caregivers coming with them during hospital visit. On the other hand, the data of eligible patients above 18 years old was acquired from themselves. Part I is aimed at collecting data on basic sociodemographic variables. Part II consisted of questions required to gather information on the prevalence, severity, and reasons of hypoglycemia, and part III contained data abstraction format, which was prepared to review the medical record of patients and assess their FBG level and BMI in the past three appointments. Maximum effort had been taken to maintain the quality of the data through the different steps like data entry, analysis, interpretation, and representation. Incomplete questionnaires were excluded while the complete questionnaires were coded and entered using Epi Data manager version 4.2.0. Then, data was exported and analyzed using Statistical Package for the Social Sciences (SPSS) version 20.0 package. Sociodemographic and clinical characteristics were summarized using frequency tables. Mean and standard deviation were calculated for continuous data. Multivariate logistic regression analysis was carried out to determine factors independently associated with hypoglycemia, and a *p* value of ≤0.05 was considered as statistically significant.

### 2.1. Ethical Statements

Ethical clearance was granted by the ethical review committee of the School of Pharmacy, Addis Ababa University, and subsequently approved by the ethical review board of SPHMMC. Prior to data collection﻿, informed consent was obtained from the patient's parents or legal guardians and from themselves for those patients under 18 years old and for those patients 18 and above years old, respectively obtained from the parents/guardians of T1DM patients under 18 years old who are included in the study and form the adult T1DM patients.

## 3. Results

### 3.1. Sociodemographic Characteristics

In this study, a total of 247 T1DM patients were involved in the data processing as one patient was excluded because of incomplete data record. Majority (54.7%) of the patients were females. More than half (52.2%) of the patients were in the age group of 5-15 years, and more than three-fourths (77.7%) of the patients had formal educational background. Regarding EDA membership, 134 (54.3%) had a membership identification card. The sociodemographic characteristics of the patients are summarized in Table 1.

### 3.2. Clinical Characteristics

Majority (61.5%) of the patents had a healthy weight while only one patient was found with obese BMI. Regarding the duration of diabetes, 54 (21.9%) had an insulin treatment history of less than one year and 130 (52.6%) have been laid between one and five years duration. Most (68%) of the patients were taking both regular and Lente insulin (NPH) while 75 (30.4%) were on Lente insulin (NPH) and 4 (1.6%) were taking only regular insulin (Table 2).

### 3.3. Prevalence and Frequency of Hypoglycemia

In this study, hypoglycemia was defined as the presence of hypoglycemic symptoms and corrected by taking carbohydrate/sweet foods. Out of the 247 patients, 233 (94.3%) of them had a history of hypoglycemia events since they were diagnosed to have T1DM. A total of 6.9 events of hypoglycemia per patient per year happened that were specifically categorized as 3.1 mild events, 2.3 moderate events, and 0.93 severe events of hypoglycemia. The frequency of the events of hypoglycemia and their category of severity are summarized in Table 3.

### 3.4. Associated Factors

**Figure 1:**
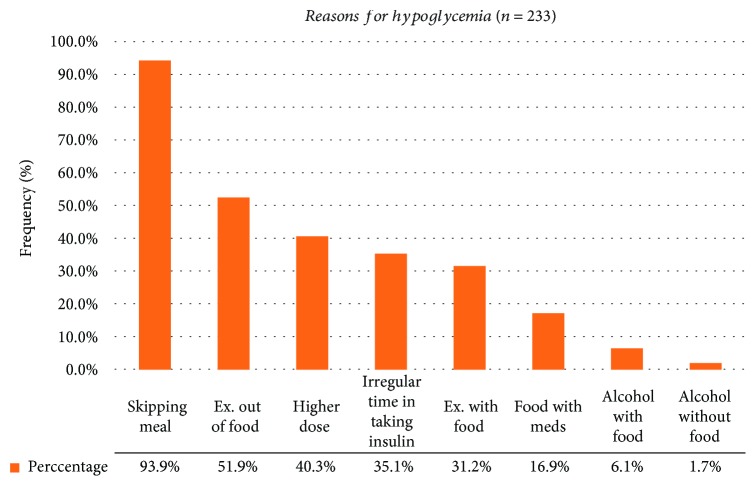
Reasons contributing to hypoglycemia among ambulatory T1DM patients attending at SPHMMC in June 01 to August 01, 2018.

Upon evaluation of the reasons contributing to hypoglycemia among the 233 respondents, majority (93.9%) experienced hypoglycemia due to skipped meal which was defined as not taking any food for two hours and above. 120 (51.9%) of them experienced hypoglycemia due to physical exercise without taking food which was defined as performing any activity without considering the routine daily activity. Drinking alcohol without taking food was found the least (1.7%) challenging reasons for the occurrence of hypoglycemia events (Figure 1). However, this value mainly belongs to adults, as most of the children did not drink alcohol.

**Figure 2:**
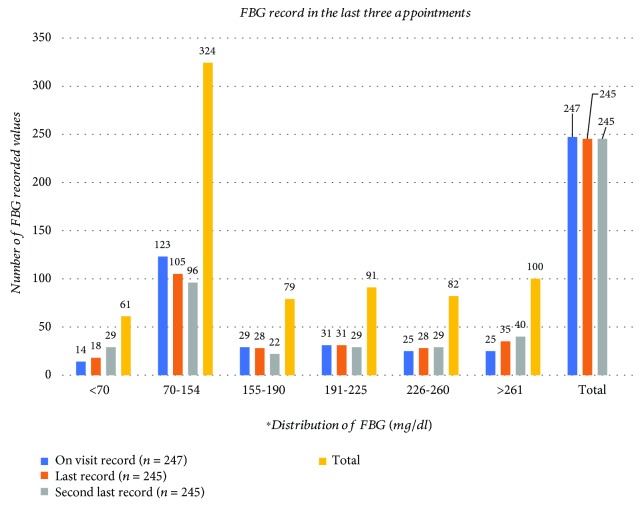
Three consecutive follow-up measurements of FBG level among ambulatory T1DM patients attending the diabetes clinics of SPHMMC from June 01 to August 01, 2018. ^∗^The FBG level of distribution category is based on DCCT, expressed as hypoglycemia: <70 mg/dl, normal: 70-154 mg/dl, excellent: 155-190 mg/dl, good: 191-225 mg/dl, fair: 226-260 mg/dl, and poor: >261 mg/dl.

**Figure 3:**
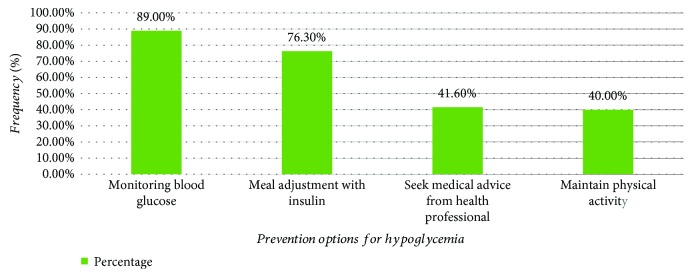
Prevention options of hypoglycemia among ambulatory T1DM patients attending the diabetes clinics of SPHMMC from June 01 to August 01, 2018.

The last three consecutive results of FBG distribution were reviewed from the chart of the study participants. Accordingly, 247 recorded values were found in the most recent visit while 245 values were found from the last two consecutive visits. A total of 61 (8.3%) recorded values were found in the hypoglycemia range. The distribution of FBG of the previous three consecutive months of the patients is summarized in Figure 2. As shown in Figure 3, blood glucose level check-up and meal time adjustment with insulin were the most prevalent prevention options of hypoglycemia; documented in 218 (89%) and 187 (76.3%) of the patients, respectively.

**Figure 4:**
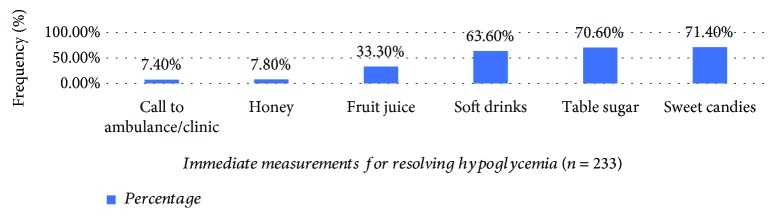
Immediate management of hypoglycemia practices among ambulatory T1DM patients attending the diabetes clinics of SPHMMC from June 01 to August 01, 2018.

The result of multiple response analysis for the immediate measurements taken for resolving hypoglycemia is summarized in Figure 4. Among the total 233 patients, taking sweet candies (71.4%) and table sugar (70.6%) were the two most common immediate measurements for resolving hypoglycemia.

In determination of the factors associated with hypoglycemia, a multivariate regression analysis was carried out using variables that had a significant association with hypoglycemia (*p* ≤ 0.05) in bivariate analysis. The variables tested in the multivariate analysis were sex, age duration of diabetes history, and blood glucose monitoring options. Results of the multivariate analysis are shown in Table 4.

## 4. Discussion

Mostly, it is impossible to eliminate hypoglycemia from the lives of T1DM patients [21]. Indeed, our result indicated that about 94.3% of the respondents experienced hypoglycemia ever since they were diagnosed with T1DM, which is comparable with the findings in the USA and Scotland [13, 16]. This showed that hypoglycemia is a challenge to T1DM patients independent of time and difference in provision of health care services. However, the finding in this study was greater than the results found in Taiwan (36.5%), South Africa (56%), and Nigeria (65%) [17–19]. The possible explanation could be due to neglected and underreported occurrence of hypoglycemia in relation to other acute complications [2, 12]. Furthermore, our study supported the report from ADA workgroup on hypoglycemia, which suggested that exclusion or inclusion criteria of studies could affect the magnitude of hypoglycemia [22]. Taken together, the variation in the prevalence of hypoglycemia episode could be due to difference in awareness about hypoglycemia among participants of the different studies.

Regarding the severity of hypoglycemia, the present study showed that nearly 7 events of mild to severe episodes of hypoglycemia occurred per patient per year. Specifically, 0.93 events of severe hypoglycemia per patient per year were reported. This finding was consistent with previous studies, which reported at least one episode per patient per year. Similarly, in Denmark, 0.4 episodes of severe hypoglycemia per patient per year were reported [23–26]. Moreover, it was comparable to the annual prospective survey conducted in Scotland, which found 8 episodes of mild hypoglycemia and 0.98 episodes of severe hypoglycemia per patient per year [27]. Although the occurrence of severe hypoglycemia was a small fraction of the total hypoglycemic events, the estimation of this incident was most reliable since severe hypoglycemia events were better documented [28]. It was impossible to prevent all the severe hypoglycemic reactions in diabetic patients. However, encouragement and education of patients and/or caregivers regarding the severity of hypoglycemia would have better outcome.

The recurrence of hypoglycemia events in T1DM patients was reported due to several reasons. In this survey, failure to observe meal timetable is the possible reason for frequent hypoglycemia in most of the patients, which is similar to reports in other studies [6, 29, 30]. However, one study in Ethiopia reported that insulin was the main reason for almost 70% of the occurrence of hypoglycemia that is higher than the result of this study [31]. Temporarily increasing glycemic targets, reducing preexercise insulin, and consuming appropriate amounts of carbohydrate may counter this. On top of that, education regarding all aspects of diabetes care is important in the prevention and treatment of hypoglycemia. Reducing hypoglycemia will involve patient empowerment and anticipatory guidance by both patients and health care providers [12].

Multivariate logistic regression analysis showed that the duration of diabetes and blood glucose monitoring options were found to be significantly associated with events of hypoglycemia. Despite its significance, the odds of suffering episodes of hypoglycemia since they were diagnosed to have T1DM were about 35% less likely in male patients compared to female patients. This result was consistent with the finding documented in the study done in Ethiopia [32]. Physiologically, there is a large sexual dimorphism in counterregulatory responses to hypoglycemia. It has been clearly demonstrated that women with T1DM have reduced neuroendocrine, autonomous nervous system, and metabolic (endogenous glucose production) counterregulatory responses compared to age and BMI matched men [33, 34]. Therefore, the present study shares that female patients might be more prone to hypoglycemia episodes due to lower counterregulatory responses to hypoglycemia than male patients.

Age was the other variable that had an association with hypoglycemia in the present study. The odds of experiencing hypoglycemia were about 8% more in patients found in the range of 5 to 15 years old compared to those greater than 30 years old. Although it was not a significant association, this implies that older patients were less likely to suffer hypoglycemic episodes compared to younger patients. Parallel with the study, a survey in the United Kingdom showed that a decrement of hypoglycemia risk occurred in patients above 60 years old [35]. However, results which were observed from studies done among diabetic patients in the USA and Korea showed that older patients suffered more with hypoglycemia compared to younger patients [7, 20]. Though our finding could not lead to a causal inference on the association of age with events of hypoglycemia, it is possible that younger patients were more susceptible to the adverse effects of neuroglycopenia. Other potential factors at this age include the irregular and often difficult eating habits of young children, the sporadic nature of their exercise habits, their inability to identify and alert caregivers about hypoglycemic symptoms, and smaller dose of insulin leading to relatively large increases when errors in dosage are made [6].

On the other hand, patients in the range of fifteen to thirty years were protective towards occurrences of hypoglycemia. This study reinforced the suggestion of recurrent and severe episodes of hypoglycemia in adolescence. Because of hypoglycemic fearfulness and emotional morbidity both of patients and their parents, these could act as a limiting factor in the achievement of good glycemic control [36]. Furthermore, aging modifies cognitive and counterregulatory hormonal responses to hypoglycemia [37]. Nevertheless, the effect of aging on increased risk of unawareness or severe episodes of hypoglycemia has also been recognized.

Duration of diabetes was the other variable that had significant association with prevalence of hypoglycemia in the present study. Indeed, patients who had a prolonged diabetes history were more likely to suffer with hypoglycemia compared to those who had short diabetic history. Like other evidences, this finding described as history of diabetes and events of hypoglycemia went almost parallel in patients' life. Previous studies had demonstrated that patients with more than five years of diabetes history were more likely to experience hypoglycemia events [2, 5, 20, 32, 38]. Blood glucose monitoring options had also a significant association with the events of hypoglycemia. Our study found that participants who monitored their blood glucose level at home in addition to their regular follow-up reported almost five and a half times of hypoglycemia events compared to those who monitored on follow-up only. This agreed with the DCCT, which verified that intensive management of diabetes (four or more times a day) is significantly associated with hypoglycemia. On the other hand, events of hypoglycemia were less likely reported in patients who had home care health professional compared to those who monitored by themselves. However, monitoring blood glucose level at home did not give a guarantee for decreasing the events of hypoglycemia. It could merely be a means of identifying daily hypoglycemic events as well as allowing immediate modification of therapeutic regimens for tightened glycemic control [1].

Regarding the limitation of the study, most importantly, the cross-sectional nature of the study design prevents us from drawing causal inferences about the relationship between the chosen covariates and outcome variables. Besides, during categorizations of the severity of hypoglycemia, the three-month data was extrapolated to a year, which may be unreliable since data were self-reported, and subject to recall bias. Similarly, other researchers recognized that the interview used for assessing the prevalence of hypoglycemia by its clinical definition may underestimate or overestimate hypoglycemia episodes and its severity when compared to other conventional objective methods such as consecutive blood glucose measurement as the events occurred. However, this study gave some useful insight into the magnitude of hypoglycemia among the study population and provided us useful baseline information for consultative, comparative, and future research purposes.

## 5. Conclusions

In conclusion, we found that hypoglycemia is still a challenge in T1DM as more than 90% of the patients who attended the outpatient diabetes clinics of SPHMMC experienced hypoglycemia since they were diagnosed. Skipping meal, doing physical exercise without taking food, and inappropriate dose of insulin were found to be the most common reasons for the recurrent episodes of hypoglycemia. Although patients suffered with all types of hypoglycemia events, severe hypoglycemia happened infrequently. Female sex, younger age, prolonged duration (>5 years) of diabetes, and frequent monitoring of blood glucose level were associated with more reporting of hypoglycemia events. Further studies should provide a special consideration to minimize, if possible to avoid, the occurrence of hypoglycemia in T1DM patients as it has been substantially high including in this study. Health care providers who are engaged in diabetes care as well as patients and/or caregivers should be educated and motivated to share their experience regarding the prevention and immediate management of hypoglycemia.

## Figures and Tables

**Table 1:** Sociodemographic characteristics of ambulatory T1DM patients who attended the diabetes clinics of SPHMMC from June 01 to August 01, 2018.

Variables	Subcategories	Total number of respondents (*N* = 247)	
		Frequency	Percent
Sex	Male	112	45.3
	Female	135	54.7

Age (years), mean ± SD	(16 ± 2.5)		
	5-15	129	52.2
	16-30	65	26.3
	>30	53	21.5

Educational background	No formal education	55	22.3
	Primary (1-8)	140	56.7
	Secondary (9-10)	38	15.4
	College & above	14	5.7

Marital status	Single	177	71.7
	Married	65	26.3
	Divorced	4	1.6
	Widowed	1	0.4

EDA membership	Yes	134	54.3
	No	113	45.7

SD: standard deviation; EDA: Ethiopian Diabetes Association.

**Table 2:** Clinical characteristics of ambulatory T1DM patients attending the diabetes clinics of SPHMMC from June 01 to August 01, 2018.

Variables	Frequency	Percent
Body mass index (BMI) ^∗^(mean ± SD (16 ± 2.8)) ^∗∗^(mean ± SD (19 ± 1.7))		
Underweight	51	21.1
Healthy weight	151	61.5
Overweight	43	17.0
Obese	1	0.4
Duration of diabetes since diagnosis (mean ± SD (4.8 ± 2.1))		
<1 year	54	21.9
1-5 years	130	52.6
>5 years	63	25.5
Types of insulin taken		
Regular insulin	4	1.6
NPH	75	30.4
Both (mix tared)	168	68.0

^∗^BMI for respondents of age ≤ 20 years was done based on the WHO BMI category as percentile adjustment. ^∗∗^BMI for respondents of age ≥ 20 years was taken the average three measurement from the medical records.

**Table 3:** Frequency of hypoglycemia events and their severity distribution in the last three months among ambulatory T1DM patients attending the diabetes clinics of SPHMMC from June 01 to August 01, 2018.

Total number of respondents (*N* = 233)				
Episodes of hypoglycemia in the last 3 months	Severity			Total
	Mild^a^ (%)	Moderate^b^ (%)	Severe^c^ (%)	
One time (*n* = 48)	18 (37.5)	30 (62.5)	*NH* ^∗^	48
Two times (*n* = 42)	52 (61.9)	27 (32.1)	05 (6.0)	84
Three times (*n* = 27)	42 (51.9)	30 (37.0)	9 (11.1)	81
Four times (*n* = 21)	38 (45.2)	33 (39.3)	13 (15.5)	84
Five times and above (*n* = 16)	32 (30.2)	47 (44.3)	27 (25.5)	106
Total episodes (*n* = 154)	182 (45.2)	167 (41.4)	54 (13.4)	403
Event rate of hypoglycemia episodes per total respondents-year (*N* = 233)	**3.1**	**2.9**	**0.93**	**6.9**

^∗^NH: not happened. ^a^Mild hypoglycemia: an event with little or no interruption of activities and no treatment assistance needed. ^b^Moderate hypoglycemia: an event with some interruption of activities but no assistance needed to administer the resuscitative actions. ^c^Severe hypoglycemia: an event requiring assistance of another person due to loss of consciousness (seizure and coma) to actively administer the resuscitative/corrective actions.

**Table 4:** Multivariate logistic regression analysis results of factors associated with hypoglycemia events among ambulatory T1DM patients attending the diabetes clinics of SPHMMC from June 01 to August 01, 2018.

Variables	Hypoglycemia (*N* = 247)		COR (95% CI)	AOR (95% CI)
	Yes (%)	No (%)		
Sex				
Male	104 (92.9)	8 (7.10)	0.60 (0.20-1.79)	0.65 (0.20-2.14)
Female	129 (95.6)	6 (4.4)	1.00	1.00
Age				
5-15 years	123 (95.3)	6 (4.7)	1.23 (0.30-5.11)	1.08 (0.12-10.01)
16-30 years	60 (92.3)	5 (7.7)	0.72 (0.16-3.16)	0.79 (0.16-3.88)
>30 years	50 (94.3)	3 (5.7)	1.00	1.00
Duration of DM (year)				
<1	49 (90.7)	5 (9.3)	0.16 (0.02-1.40)	0.09 (0.01-0.90)^∗^
1-5	122 (93.8)	8 (6.2)	0.25 (0.03-2.01)	0.15 (0.02-1.32)
>5	62 (98.4)	1 (1.6)	1.00	1.00
Blood glucose monitoring options				
Self/family+RFU^£^	147 (96.7)	5 (3.3)	3.01 (0.95-9.53)	5.77 (1.16-28.66)^∗^
HCP^¥^+RFU	8 (88.8)	1 (8.3)	0.82 (0.09-7.42)	1.27 (0.13-13.02)
RFU only	78 (91.9)	8 (8.1)	1.00	1.00

^∗^Statistically significant (*p* value ≤ 0.05). HCP^¥^: health care professional; RFU^£^: regular follow-up.

## Data Availability

The dataset which supported our findings is available from the primary author (HK).

## Abbreviations

ADA: American Diabetes Association
DCCT: Diabetes Control and Complication Trial
EDA: Ethiopian Diabetes Association
FBG: Fasting blood glucose
IDF: International Diabetes Federation
ISPAD: International Society for Pediatrics and Adolescent Diabetes
SPHMMC: St. Paul's Hospital Millennium Medical College
SMBG: Self-monitoring of blood glucose
T1DM: Type 1 diabetes mellitus.
